# Autoimmune glial fibrillary acidic protein astrocytopathy suspected of intracranial infection: A case report

**DOI:** 10.1016/j.idcr.2025.e02303

**Published:** 2025-06-24

**Authors:** Weijia Li, Yuan Lin, Tan Xu, Mamatili Xilifu

**Affiliations:** a Department of Intensive Care Unit, Peking University Shenzhen Hospital, No. 1120, Lianhua Road, Futian District, Shenzhen 518000, China; b Department of Cardiology, Peking University Shenzhen Hospital, No. 1120, Lianhua Road, Futian District, Shenzhen 518000, China; c Department of Intensive Care Unit, Kashgar People's Hospital, No. 80, Chaoyang Road, Xinjiang, Kashi Prefecture 844000, China

**Keywords:** Intracranial infections, GFAP-A, mNGS, Plasma replacement, Case report

## Abstract

Autoimmune glial fibrillary acidic protein astrocytopathy (GFAP-A) is an autoimmune-mediated neurological disease with cerebrospinal fluid GFAP-IgG as a marker. This is a rare disease. This paper reported a GFAP-A patient suspected intracranial infection whose diagnosis and treatment were summarized, and the value of negative metagenomic second-generation sequencing (mNGS) results for the exclusion of infectious diseases and the preferred treatment plan for GFAP-A were discussed.

## Introduction

GFAP-A is a new type of autoimmune inflammatory disease of central nervous system that has been explored by doctors in recent years.In 2016, the Mayo Clinic professor Lennon’s team for the first time reported 16 cases of GFAP-A clinical symptoms and imaging findings, including inflammatory meningitis, encephalitis and myelitis, and pointed out that anti-GFAP antibody is the specific biological markers of the disease [1], [2], [3], [4], [5], [6]. GFAP-A is a very special disease, the etiology and pathogenesis are not clear at present. Most researchers demonstrate that it may be related to tumor, virus infection, similar to autoimmune encephalitis [6]. GFAP-A is often misdiagnosed as intracranial infection because of its complex and diverse clinical manifestations. Therefore, the early detection, accurate diagnosis, and timely and effective treatment are the key to improve the cure rate.

## Case presentation

A 28-year-old young female patient was admitted to the hospital for "fever for 3 days". She developed fever after catching cold 3 days ago, with a maximum body temperature of 39.5℃, accompanied by headache, dizziness, chills, nausea and vomiting. After taking anti-fever drugs, she still had fever and came to the emergency department of our hospital. Emergency blood routine indicated "leukocytes 11 * 10e9 / L, neutrophil 9.6 * 10e9 / L", and urine routine indicated "white blood cells 5–10 / HP, occult blood 1 +", with no abnormality on chest CT. The patient was admitted to the emergency ward for further treatment. After admission, urinary tract infection was initially considered, and cefoperazone was given for anti-infection.Because the patient had headache and neck resistance, the possibility of nervous system infection was not ruled out, so ceftriaxone was used to treat the infection. On admission day 1, we perfected the head CT and no significant abnormalities was found. Lumbar puncture was performed to obtain cerebrospinal fluid(CSF)for examination(Culture + Biochemistry + mNGS).On the second day of admission, the patient presented with restlessness and delirium, CSF biochemistry (Table 1) suggested intracranial bacterial infection. CSF mNGS results showed no pathogenic microbial fragments. CSF culture suggested *Staphylococcus cephalus* infection. The antibiotics were adjusted as ceftriaxone + vancomycin + acyclovir according to the neurology consultation opinion, and transferred to the neurology department. On day 3 after admission, the patient gradually developed dyspnea, decreased muscle strength in all limbs, and coma. Arterial blood gas analysis suggested PaCO_2_ 80 mmHg, considered acute type II respiratory failure with pulmonary encephalopathy.The patient was then transferred to the ICU for advanced life support.

**Table 1:** Changes in CSF routine and biochemical indicators during treatment date.

	2nd	6th	10th	12th	17th	25th	36th
Total cell count (E + 6/L)	586	376	221	112	127	158	97
leukocyte (E + 6/L)	411	191	191	40	21	58	43
Total protein (g/L)	7.73	3.90	2.83	1.47	0.83	0.41	0.31
glucose (mmol/L)	2.14	1.91	2.75	2.79	4.08	3.69	2.59

**Fig. 1:**
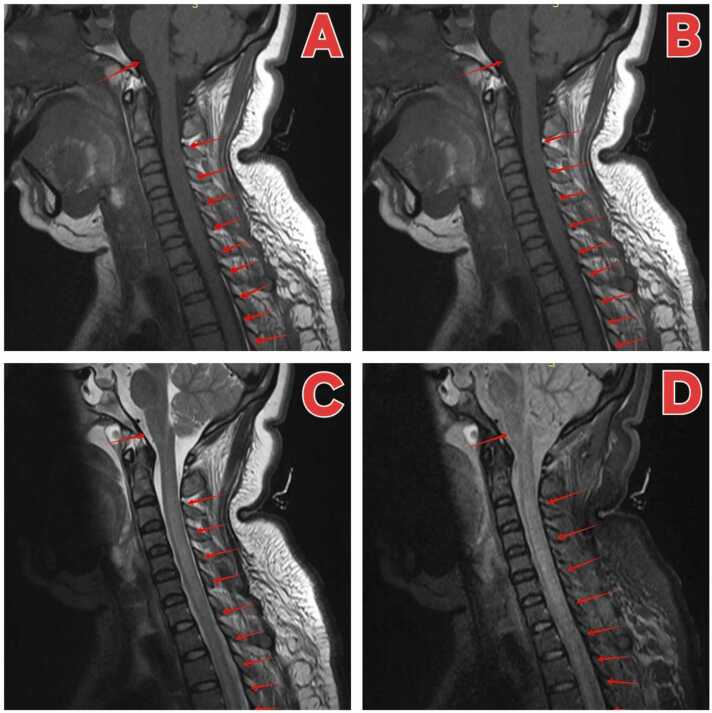
Image(Head and spinal cord MR) findings at the admission. A)sagittal image of plain T2-weighed MRI, B) sagittal image of plain T2-weighed MRI, C) sagittal image of plain T1-weighed MRI,D) sagittal short tl inversion recovery(STIR) MR image.Red arrowhead: original cerebral groove lesion showed no clear indication, linear enhancement of medulla surface, considering infection; abnormal signal of cervical cord and thoracic cord with enhancement, consistent with myelitis.

After ICU admission, acute meningoencephalitis was still diagnosed (*Staphylococcus cephalus*). After mechanical ventilation, the patient was treated with an anti-infection regimen, and CSF was reviewed on the sixth day (Table 1), which showed that the intracranial infection improved. On the ninth day after admission, the patient was conscious, but the muscle strength of all limbs continued to decline, the muscle strength of the proximal extremities was grade 0 and grade 1, and the muscle tone of both lower limbs was low, head + spinal cord MR examination: original cerebral groove lesion showed no clear indication, linear enhancement of medulla surface, considering infection; abnormal signal of cervical cord and thoracic cord with enhancement, consistent with myelitis (Fig. 1 A–D).Considering the condition deteriorated again, CSF was taken again for mNGS, the results still showed no pathogen gene fragments which indicated a high probability of non-infectious disease.

On the 10th day of admission, CSF was tested for aquaporin 4 (AQP 4) antibody, myelin oligodendrocyte glycoprotein antibody (MOG), glial fibrillary acidic protein (GFAP) antibody, and myelin basic protein antibody (MBP). On the 12th day of admission, GFAP antibody was 1:100 positive (negative reference value). GFAP-IgG, as an important biomarker of autoimmune encephalitis, is mainly detected by live cell immunofluorescence assay (L-CBA) in Kingmed Diagnostics. So we revised the diagnosis of " autoimmune glial fibrillary acidic protein astrocytopathy "

On the 12th day of admission, methylprednisolone was given 1000 mg/d for shock treatment, and CSF was taken again for examination. After three days of continuous use, the dosage was gradually reduced, and the transition to oral hormone therapy was made. Meanwhile, symptomatic treatment such as gamma globulin (0.4 g/kg.d for 5 days), anti-infection and rehabilitation were given. Patients slightly improved, upper limb muscle strength can gradually recover to level 3–4, lower limb muscle strength is still level 0–1, although repeated review CSF prompt improvement (Table 1), but the patient again repeated high fever. On the 30th day after admission, methylprednisolone was given 1000 mg/d shock treatment again, and the condition did not improve significantly. On the 33rd day after admission, the patient was treated with plasma exchange daily. The patient's body temperature gradually returned to normal, and the immunosuppressive agent mortemycophenol ester was taken orally at the same time. After 5 times of plasma exchange, the patient was successfully removed from the ventilator, and the muscle strength of the upper limb recovered to grade 4–5, while the muscle strength of the lower limb was still grade 0–1. On the 37th day, the patient was transferred to the general ward of the department of Neurology, and continued to undergo enhanced consolidation therapy with plasma exchange. On the 46th day, the GFAP antibody was reviewed at 1:32 (lower than before), and the patient was discharged with improvement.

After discharge, the patient's compliance was good, and after several months of rehabilitation training, the lower limb muscle strength gradually improved, and the lower limb muscle strength of the patient was restored to grade 4 at the last follow-up.

## Discussion

In this case, the patient presented with fever, headache and elevated infection indicators,meningeal irritation sign positive, CSF examination and imaging examination positive,which point to intracranial infection.However, there are many uncertain factors: poor anti-infection effect, no obvious brain parenchymal lesions but long segmental myelitis, etiological results except the first CSF culture showed positive *Staphylococcus capitis*, and repeated CSF culture and blood culture were negative, and two CSF mNGS showed negative results. The mNGS is a diagnostic technology for high-throughput sequencing and comprehensive analysis of microbial and host genetic material (DNA and RNA) in samples, which shows great advantages in diagnosing complex and severe infections [7]. According to the literature, the positive detection rate of CSF by mNGS reached 55 %, which was higher than that of the traditional detection method. A study showed that for the CSF diagnosis of meningitis and encephalitis of infectious etiology, compared with the traditional clinical laboratory examination results, the mNGS’s sensitivity is 73 %, specificity is 99 %, positive predictive value of 81 %, negative predictive value as high as 99 % [8], proved that mNGS negative to exclude infectious diseases has considerable reliability. In this case, conventional pathogen examination and two mNGS results are negative, gave us quite important diagnostic ideas, basically excluded intracranial infection, need to consider other possible non-infectious diseases such as neuromyelitis optica spectrum disorder (NMOSD), multiple sclerosis (MS), and autoimmune encephalitis, etc.NMOSD is characterized by positive AQP4 antibody, long-segment myelitis (≥ 3 vertebrae) and optic neuritis, and is diagnosed by AQP4 detection and spinal cord MRI. MS is manifested as positive oligoclonal bands in cerebrospinal fluid, periventricular/near-cortical lesions, and a recurrence-remission course. GFAP-A is positive for anti-GFAP antibodies, accompanied by linear enhancement around the ventricles and symptoms of meningeal/encephalitis/myelitis, often accompanied by tumors. Antibody detection and tumor screening are required [4], [6], [9], [10], [11]. Carrying out CSF detection against GFAP antibody, eventually patient diagnosed.

GFAP-A is very rare in our clinical work, and only advanced clinicians can associate it with this disease, which often leads to the delayed diagnosis and treatment of the disease. Secondly, the early clinical manifestations of the patient are like intracranial infection, such as fever, headache, consciousness disorder, etc. Magnetic resonance imaging also indicates enhanced meninges, and cerebrospinal fluid shows light white blood cells and elevated protein. So it is easy to be misdiagnosed as bacterial, viral, tuberculous meningoencephalitis, infectious diseases, etc. [4], [9]. The same is true for this case. In the early stage, we diagnosed bacterial meningitis and treated it with anti infection therapy. However, the number of cells and protein in cerebrospinal fluid decreased, and there were still repeated high fever, and the muscle strength decreased significantly. The patient soon presented with clinical manifestations of long-term myelitis, which cannot be simply explained as meningitis. Fortunately, after standard diagnosis and differential diagnosis analysis, we finally confirmed "GFAP-A" this a rare disease diagnosis.

Compared with the existing literature reports, we summarize the following diagnostic points: 1. The etiology is not clear, and may be related to infection and tumor; 2. The clinical manifestations are not specific, the early symptoms include fever, runny nose, cough, etc. With the development of the disease, the clinical syndrome of meningoencephalitis and myelitis; 3. The typical radial enhancement and (or) of enhanced MRI. 4. Positive GFAP antibody in cerebrospinal fluid. Among them, the positive antibody of GFAP has high specificity and sensitivity and is a specific biological marker of this disease, which has been confirmed by clinical studies and animal experiments [12], [13], [14]. The detection of GFAP is based on the positive titer of cerebrospinal fluid [6], which is generally higher than that of blood samples, and only when the blood sample is positive should be combined with clinical practice [3], [4], [6], [10].

Currently, there is no standard treatment plan for GFAP-A. The acute treatment includes high-dose methylprednisolone shock therapy, intravenous gamma globulin shock therapy and plasma exchange, and long-term treatment includes oral hormones and immunosuppressants [2], [6]. In this paper, after the diagnosis of the patient was confirmed, we also standardized high-dose steroid hormone shock therapy (methylprednisolone 1000 mg intravenous injection for 5 days) and intravenous immunoglobulin injection, and the condition was also improved to a certain extent, the muscle strength of the upper limb and respiratory muscle was restored to a certain extent, and tracheotomy was avoided. Existing literature reports conclude that approximately 70 % of patients respond well to hormone therapy. However, during the transition from intravenous hormone therapy to long-term oral hormone therapy, we found that although the limb muscle strength of the patient improved, the upper limb muscle strength gradually recovered to grade 3–4, and the lower limb muscle strength recovered to grade 0–1, however recurrent high fever occurred again, indicating poor response to hormone therapy, which was inconsistent with the above literature reports [4], [5], [11], [15]. After repeated plasma exchange and standardized hormone and immunosuppressive therapy, the patient's body temperature quickly returned to normal, the patient quickly got off the ventilator, the upper limb muscle strength recovered to grade 4–5, the cerebrospinal fluid GFAP antibody titer dropped to grade1:32, and finally improved and was discharged. At present, due to the small number of cases of this disease, plasmapheresis is rarely used in the treatment of acute stage cases, and there is no literature support for whether plasmapheresis can be used as the first line therapy. Meanwhile, we need to emphasize that the decrease in GFAP-IgG antibody titer during the treatment process mainly reflects the effectiveness of immunotherapy and the reduction in disease activity, and it is an important reference for prognosis evaluation. In the later stage of the follow-up, we learned that the patient's muscle strength recovered, the MRI reexamination basically returned to normal, and there was no recurrence of the disease [11], [13], [14]. Most patients have a good prognosis, a few patients have poor response to treatment or even die, and some patients may be left with different degrees of functional disability [10], [11]. The disease is also prone to recurrence, especially in the process of hormone reduction 1–2 years after the onset [15], and doctors and patients should have full awareness of it.

## Conclusions

In summary, for patients suspected of encephalitis or encephalomyelitis, when conventional pathogen culture and mNGS are negative and empirical anti-infective therapy is ineffective, we should fully understand the significance of negative mNGS results for the exclusion of infectious diseases and the possibility of GFAP-A should be considered. Meanwhile, plasma exchange may be the preferred treatment for the acute phase of GFAP-A.

## Author Agreement

All signatories have seen and approved the final version of the submitted manuscript. We guarantee that this article is the author's original work and has not been published in advance or considered for publication elsewhere.

## CRediT authorship contribution statement

**Mamatili Xilifu:** Writing – review & editing, Writing – original draft, Visualization, Validation, Supervision. **Li weijia:** Writing – review & editing, Writing – original draft, Visualization, Validation, Supervision, Software. **Tan Xu:** Writing – original draft, Formal analysis, Data curation, Conceptualization. **Yuan Lin:** Writing – original draft.

## Ethical approval

This study was approved by the Clinical Research Review Committee of Peking University Shenzhen Hospital. (2025-EC-PKUSZH-CASE-014).

## Consent

Written informed consent was obtained from the patient.

## Funding source

Not applicable.

## Declaration of Competing Interest

The authors declare that they have no known competing financial interests or personal relationships that could have appeared to influence the work reported in this paper.

## Data Availability

The datasets used and/or analyzed during the current study are available from the corresponding author on reasonable request.
